# Development of novel DNA vaccine for VEGF in murine cancer model

**DOI:** 10.1038/srep03380

**Published:** 2013-11-29

**Authors:** Mariko Kyutoku, Hironori Nakagami, Hiroshi Koriyama, Hideki Tomioka, Futoshi Nakagami, Munehisa Shimamura, Hitomi Kurinami, Pang Zhengda, Dong Hyun Jo, Jeong Hun Kim, Nobuyuki Takakura, Ryuichi Morishita

**Affiliations:** 1 Department of Clinical Gene Therapy, Osaka University Graduate School of Medicine; 2 Division of Vascular Medicine and Epigenetics, Department of Child Development, United Graduate School of Child Development, Osaka University, Kanazawa University, Hamamatsu University School of Medicine, Chiba University and Fukui University; 3 Department of Geriatric Medicine, Osaka University Graduate School of Medicine; 4 Fight against Angiogenesis-Related Blindness (FARB) Laboratory, Clinical Research Institute, Seoul National University Hospital, Seoul, Republic of Korea; 5 Department of Biomedical Sciences, College of Medicine, Seoul National University, Seoul, Republic of Korea; 6 Department of Ophthalmology, College of Medicine, Seoul National University, Seoul, Republic of Korea; 7 Department of Signal Transduction, Research Institute for Microbial Diseases, Osaka University, Suita, Osaka, Japan; 8 JST(Japan Science and Technology Agency), CREST, Tokyo, Japan

## Abstract

We developed DNA vaccine for vascular endothelial growth factor (VEGF), which may provide the therapeutic option instead of anti-VEGF antibody, bevacizumab. Plasmid containing VEGF mini-gene was constructed in the insertion of B-cell epitope of Hepatitis B core protein [HBc-VEGF], which was an epitope carrier. High titer of anti-VEGF antibody was observed in BALB/c mice which were intramuscularly immunized with HBc-VEGF by electropolator. In mice inoculated with colon 26 cells, tumor volume and microvessel density was decreased in HBc-VEGF with a significant prolonged survival. Co-treatment of purified IgG from immunized mice with HBc-VEGF showed *in vitro* neutralizing activity for VEGF-induced ERK phosphorylation and tube formation in cultured endothelial cells. Furthermore, intravitreally injection of this purified IgG reduced the neovessel formation in the mouse oxygen-induced retinopathy and laser-induced choroidal neovascularization models. These results first provided that DNA vaccine against VEGF possessed the anti-angiogenic effect, leading to prolonged survival in mouse cancer model.

Angiogenesis is a critical process in the invasion, growth and metastasis of most solid tumors. Anti-angiogenic therapy for targeting the tumor vasculature has been recently used in various cancer patients, as the function of vascular endothelial growth factor (VEGF)-family ligands and their receptors is critical in tumor angiogenesis^1^,^2^,^3^. For example, bevacizumab, a humanized anti-VEGF monoclonal antibody, demonstrated the therapeutic benefits to treat various malignancies including metastatic or advanced colorectal cancer^4^, non-small cell lung cancer^5^, metastatic breast cancer^6^, glioblastoma^7^ and metastatic renal cancer^8^. Of importance, bevacizumab prolonged the survival of the patients with advanced colon cancer when combined with triple chemotherapy^9^,^10^. Also, aflibercept, as known as VEGF Trap, is a soluble chimeric decoy receptor for VEGF that fuses the portions of human VEGFR1 and VEGFR2 with Fc region of human IgG1, binds all isoforms of VEGF-A, VEGF-B and PlGF^11^. Aflibercept has shown the potent anti-tumor effects in the models of cancer and in clinical trials^12^,^13^. However, there are still several clinical problems for anti-VEGF/VEGFR antibodies such as the high cost and the inconvenience of monthly or repeated injections throughout the life.

Alternatively, the vaccines for VEGF might be attractive, due to the relatively low cost and less treatment regime. Among vaccines, DNA vaccines for VEGF or its receptor have been tried in different preventive and therapeutic strategies against tumor. Niethammer et al. showed that oral DNA vaccine encoding full-length VEGFR-2 effectively protected mice from lethal challenge with melanoma, colon carcinoma, lung carcinoma cells and reduced metastases in therapeutic model^14^. The vaccination induced CTL-mediated killing of endothelial cells as well as producing antibody for VEGFR-2. Similarly, Xie et al. also showed that immunization with human VEGFR-2 LDC (liposome-DNA complex) induced therapeutic and protective anti-tumor effects in mouse model of colon carcinoma and breast cancer^15^. However, in this strategy CTL-mediated killing of endothelial cells may induce the side effects in terms of safety issue. Therefore, we modified the vaccine to exclude T-cell epitopes from antigen, leading to avoid T-cell activation without disrupting B-cell epitopes responsible for antibody production.

In this study, we selected mini-gene from the targeted antigen, VEGF, which are the residues related to VEGF-bevacizumab/VEGFR binding, and developed DNA vaccine for VEGF using Hepatits B virus core (HBc) system. HBc protein was used as an epitope carrier to enhance the immunogenicity as previously reported^16^,^17^. The neutralizing activity of anti-VEGF antibody produced by immunization with DNA vaccine for VEGF was examined.

## Results

### Production of DNA vaccine for VEGF

**Figure 1:**
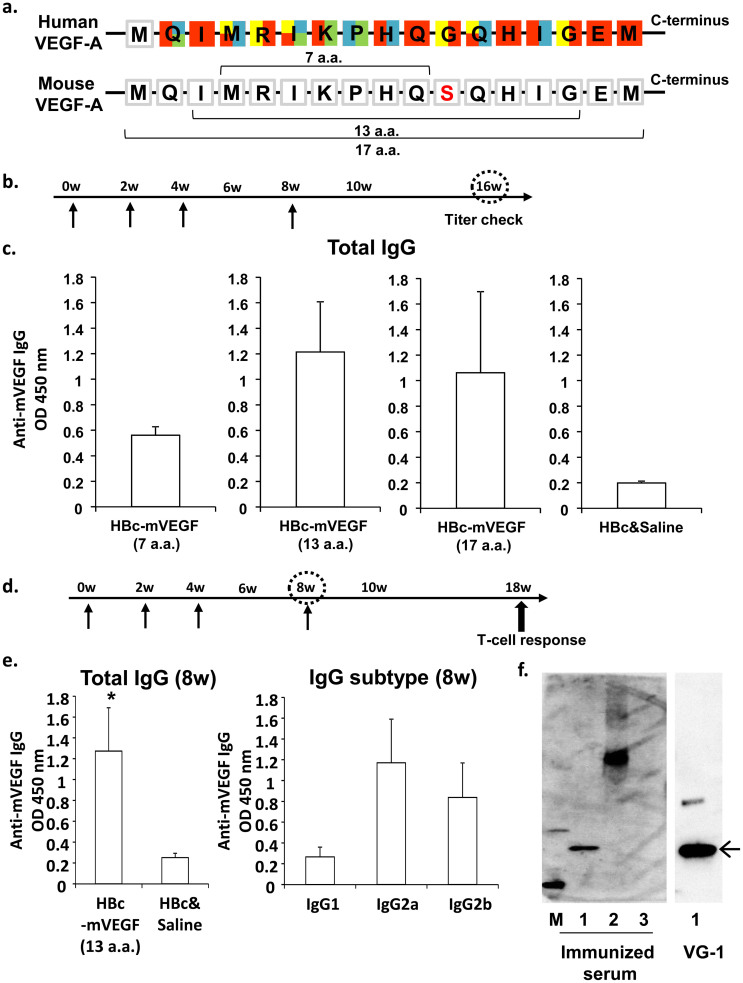
DNA vaccination for VEGF in BALB/c. (a) Framework residues at VEGF-bevacizumab interface. Red, residues in binding surface with bevacizumab; Yellow, especially important residues of Red; Blue, residues in binding surface with VEGFR-1; Green, residues in binding surface with VEGFR-2. Each residue is represented by single-letter codes. (b) and (d) Time course of DNA vaccination. Vaccination was initially performed using 6 week-old mice (0w), and subsequent vaccinations were given at 2 (2w), 4 or 16 weeks after first vaccination. (c) Titers of anti-VEGF antibodies at 16 weeks. Total IgG titers for VEGF were quantified in mouse sera (100 dilution) from mice immunized with HBc-mVEGF (7 a.a.), HBc-mVEGF (13 a.a.), HBc-mVEGF (17 a.a.) or HBc, respectively. (e) Titers of anti-VEGF antibodies at 8 weeks. Total IgG titers for VEGF were increased only in mouse sera (100 dilution) from the HBc-mVEGF (13 a.a.) group (left panel). The IgG subtype distribution (IgG1, IgG2a or IgG2b) was also evaluated using subtype-specific IgG antibodies in mouse sera (100 dilution) from the HBc-VEGF (13 a.a.) group (right panel). (f) Specific binding of immunized serum to VEGF. Immunized serum used as primary antibody in western blot bound to not only BSA-conjugated mVEGF (13 a.a.) but recombinant mouse VEGF (rmVEGF). Loading samples were as follows; lane 1, recombinant mouse VEGF-A; lane 2, BSA-conjugated mVEGF (13 a.a.); lane 3, BSA-conjugated human Angiopoietin-2 peptide as negative protein. VG-1, commercial monoclonal antibody against VEGF, was used as positive antibody. Data were means ± S.E.M. *p < 0.05 versus control (HBc and Saline).

To develop DNA vaccine for neutralizing VEGF, we selected the target antigen of VEGF, which showed the binding epitope of VEGF for the humanized anti-VEGF antibody, bevacizumab, and the binding region of VEGFR-1 or VEGFR-2 (Fig. 1a)^18^. The core candidate antigen was seven amino acids (MRIKPHQ; 7 a.a.) which overlapped the binding surface with bevacizumab, VEGFR-1 or VEGFR-2. Because only seven amino acids could be less immunogenic for vaccination, HBc protein was utilized as an epitope carrier protein which is able to self-assemble into icosahedral virus-like particles (VLPs) in heterologous expression systems. To confirm whether this DNA vaccine system would sufficiently induce anti-VEGF antibody production, BALB/c female mice were immunized with pcDNA3.1-HBc-mVEGF (7 a.a.) [HBc-mVEGF (7 a.a.)], pcDNA3.1-HBc [HBc] or saline, respectively, by intramuscular administration using electroporator, three times every two weeks and additional booster after third immunization (Fig. 1a). As a result, high titer of anti-VEGF antibody was not observed in HBc-mVEGF (7 a.a.) group compared to control (HBc and saline) group (Fig. 1b). Because this 7 a.a. sequence might not be enough for B-cell epitope to induce anti-VEGF antibody, the long sequence was also designed as a candidate antigen, which covered the binding surface with bevacizumab, VEGFR-1 or VEGFR-2. We added 6 or 10 amino acids to core sequence, and created the target 13-amino acid sequence (IMRIKPHQSQHIG; 13 a.a.) and 17-amino acid sequence (MQIMRIKPHQSQHIGEM; 17 a.a.), respectively. Then, pcDNA3.1-mVEGF (13 a.a.) [HBc-mVEGF (13 a.a.)] and pcDNA3.1-mVEGF (17 a.a.) [HBc-mVEGF (17 a.a.)] were similarly constructed and vaccinated to mice. As shown in Fig. 1c, both antigens successfully induced the production of anti-VEGF antibody. Relatively high titer of anti-VEGF was observed In HBc-mVEGF (13 a.a.) group as compared to 17 a.a. and 7 a.a groups (Fig. 1c). Thus, we decided 13 a.a. (IMRIKPHQSQHIG) as the antigen of VEGF DNA vaccine using HBc system (SI. 1a and 1b). The functionality of expression vector of HBc-mVEGF (13 a.a.) was confirmed in transfected COS-7 cells. Each construct expressed mRNA of the expected length and protein of expected molecular mass (SI. 2a).

We confirmed the titer of anti-VEGF antibody at eighth week, four weeks after third immunization (Fig. 1d), and high titer was observed in HBc-mVEGF (13 a.a.) group (Fig. 1e, 1f). In the analysis of IgG subtypes, this immunization could lead to Th1-biased immune responses with predominant IgG2a and IgG2b production (Fig. 1e). We further confirmed whether the produced serum by immunization with HBc-mVEGF (13 a.a.) would recognize the recombinant mouse VEGF-A protein. The specific binding of immunized serum with both mouse and human was shown in western blot analysis, confirmed by using the commercial antibody against VEGF, VG-1 (Fig. 1f and SI. 2b).

### Neutralizing activity of anti-VEGF antibody produced by VEGF DNA vaccination

**Figure 2:**
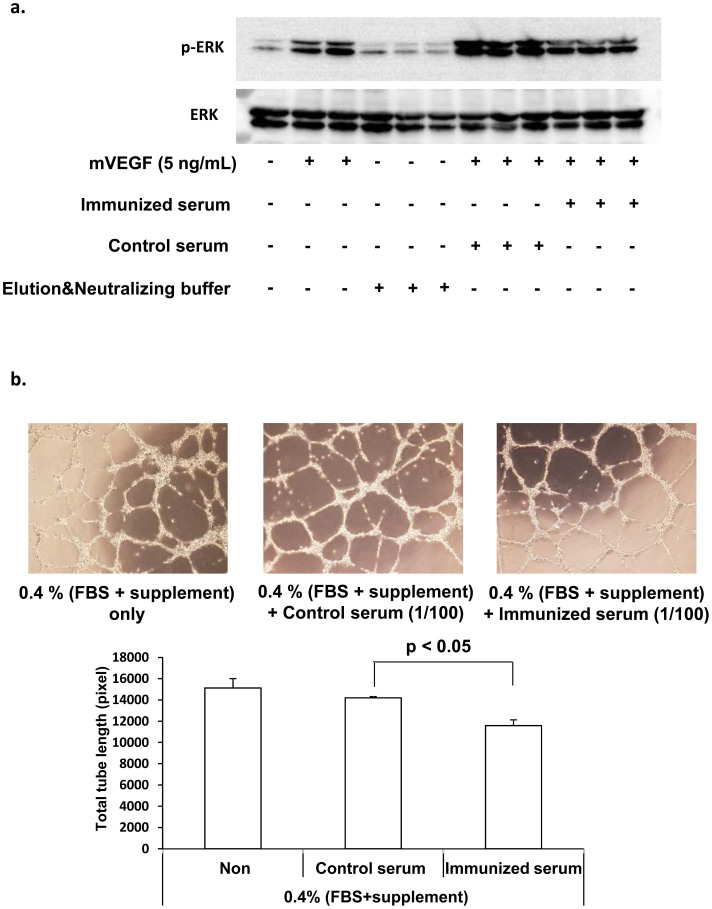
Neutralizing activity of immunized serum in HUVECs. (a) Western blot analysis of cell lysates from HUVECs stimulated for 10 min with mVEGF at 5 ng/mL in the presence of immunized serum or control serum for p-ERK and total ERK under the same experimental condition. (b) Effects of immunized serum on VEGF-induced tube formation of HUVECs. HUVECs were plated on matrigel-coated plates at density of 1 × 10^5^ cells/well and incubated in the presence of control serum or immunized serum. After 7 hours, capillary network were photographed and quantified. Representative endothelial tubes were shown. Magnification: 50×. Data were means ± S.E.M. of triplicates.

To examine the neutralizing activity of serum from vaccinated mice, we purified mouse IgG of serum using by Protein G column as described in method section (SI. 1e). Treatment of immunized serum, but not control serum, significantly attenuated VEGF-A-induced ERK1/2 phosphorylation in HUVECs (Fig. 2a and SI. 3). In the tube formation of HUVECs in matrigel, in the presence of EBM-2 with 0.4% FBS and 0.4% supplement, the addition of immunized serum also significantly attenuated the tube formation of HUVECs, as compared to the addition of control serum (Fig. 2b).

### Anti-tumor effects in tumor-bearing mice with colon 26 carcinoma cells

**Figure 3:**
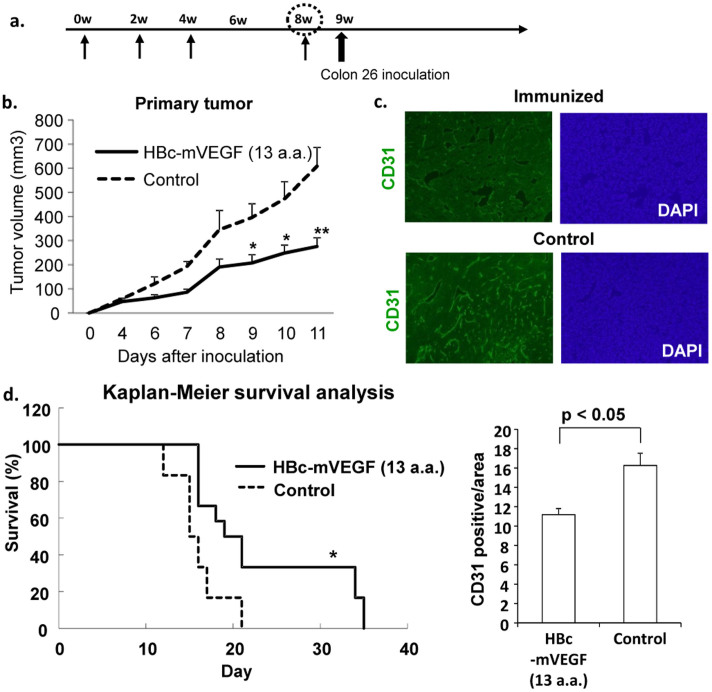
Effects of VEGF DNA vaccine in tumor-bearing mice. (a) Time course of DNA vaccination and tumor implantation. One week after last immunization, colon 26 cells were inoculated subcutaneously into mice. (b) Primary tumor volume. Tumor volume was measured by using caliper and the following formula: 0.5 × (length) × (width)^2^. HBc-mVEGF (13 a.a.) immunization inhibited the tumor growth compared with control group. (c) Immunohistochemistry staining of primary tumor tissue (CD31). Tumors were excised, sectioned, and stained with anti-CD31 antibody to visualize the blood vessels. The number of CD31-positive cells per area was counted. (d) Kaplan-Meier survival curve. *p < 0.05; **p < 0.01 versus control.

Given the neutralizing activity of the immunized serum in vitro, we injected colon 26 cells on mouse back subcutaneously one week after last immunization (Fig. 3a). In this tumor-bearing model, mice perish from cancer cachexia contributed some mediators such as IL-6, IL-1 and TNFα on day 14 to 20 after cancer cell implantation^19^,^20^. HBc-mVEGF (13 a.a.) vaccination or HBc control was intramuscularly administred using electroporator, three times every two weeks and additional booster after third immunization, similarly. The mice vaccinated with HBc-mVEGF showed a significant inhibition of primary tumor growth in early phase as compared to the mice of control group (Fig. 3b). In the microvessel density in tumor tissue, as the indication of effects of anti-angiogenic agents assessed by immunohistochemical staining for CD31, immunization with HBc-mVEGF significantly decreased CD31-positive cells, as compared to control group in the subcutaneous colon 26 tumor model (Fig. 3c). Of importance, consistent with these results, DNA vaccine for VEGF significantly improved the survival in this cancer model through the VEGF-neutralizing activity (Fig. 3d).

### In vivo anti-angiogenic effects of VEGF DNA vaccine on retinal and choroidal neovascularization

**Figure 4:**
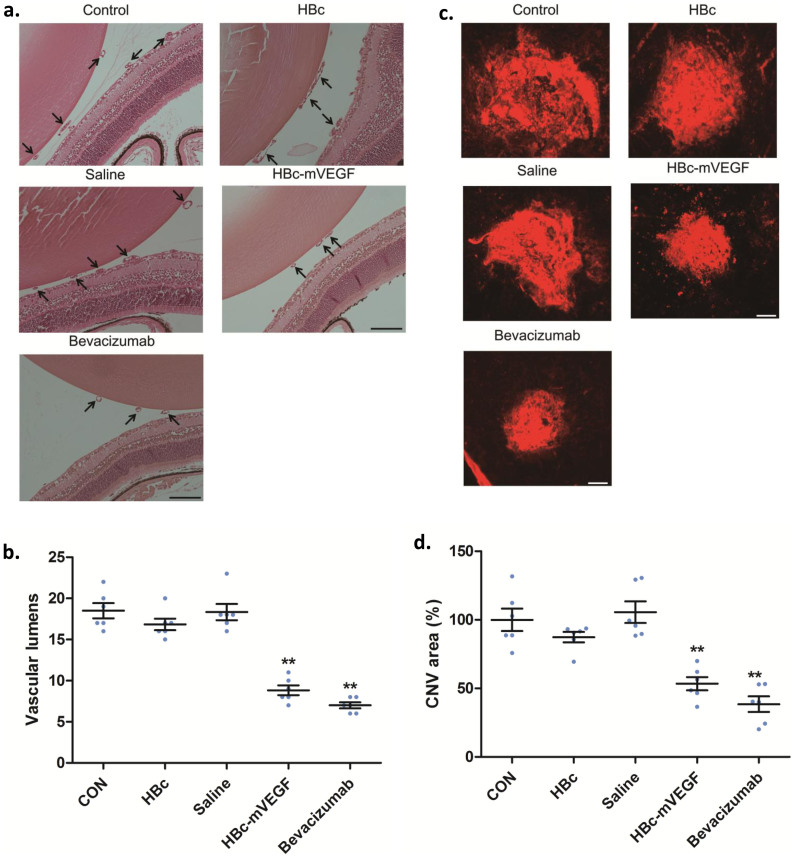
Neutralizing activity of immunized serum in retinal and choroidal neovascularization. (a) Effects of immunized serum on retinal neovascularization in mouse OIR model. Purified IgG from immunized serum was intravitreally injected into the right eyes of mice at P14 and the enucleated eyes were prepared for further analyses at P17. For qualitative analyses, FITC-dextran (500 kDa) was intravenously perfused and the retinal tissues were prepared and flat-mounted. The images from each group was intentionally modified and combined to demonstrate the inhibitory effect of the purified IgG on the formation of vascular tufts. Quantitative analysis was performed by direct counting of vascular lumens on the vitreal side of the inner limiting membrane in at least 10 sections from each eye (*n* = 6). Data were means ± S.E.M. CON, control; HBc, purified IgG from mice immunized with HBc; Saline, purified IgG from mice immunized with saline; HBc-mVEGF; purified IgG from mice immunized with HBc-mVEGF. **p < 0.01. Scale bar, 100 μm. (b) Effects of immunized serum on mouse laser-induced CNV model. CNV was induced by laser photocoagulation. Scale bar, 100 μm. Purified IgG from immunized serum was intravitreally injected into the right eyes of mice at 10 days after the photocoagulation and the enucleated eyes were prepared for further analyses after additional 4 days. Quantitative analysis was performed by measuring the CNV area in 4 burn sites from each eye (*n* = 6). Data were means ± S.E.M. CON, control; HBc, purified IgG from mice immunized with HBc; Saline, purified IgG from mice immunized with saline; HBc-mVEGF; purified IgG from mice immunized with HBc-mVEGF. **p < 0.01. Scale bar, 100 μm.

To further confirm *in vivo* neutralizing effect of purified mouse IgG of serum from immunized mice, we intravitreally injected purified mouse IgG into the eyes of mouse oxygen-induced retinopathy (OIR) and laser-induced choroidal neovascularization (CNV) models. Each model represents the pathologic conditions mimicking retinal neovascularization and choroidal neovascularization, respectively. Both conditions are known to be mainly mediated by VEGF^21^. Interestingly, the purified mouse IgG effectively reduced the formation of vascular tufts and the number of vascular lumens in the mouse OIR model (Fig. 4a and SI. 3). Furthermore, the area of CNV demonstrated by isolectin B4 staining was also significantly decreased by the treatment with the purified mouse IgG of serum from mice previously immunized with HBc-mVEGF (Fig. 4b). Particularly, anti-angiogenic and neutralizing activities of anti-VEGF antibody from the purified mouse IgG were comparable to those of commercialized monoclonal anti-VEGF antibody, bevacizumab (Fig. 4a and 4b).

## Discussion

Tumor angiogenesis is a crucial process in tumor development. Among the angiogenic factors, VEGF-A is the major player both in physiological angiogenesis and in pathological angiogenesis. This recognition has led to VEGF-targeted approaches, the development of several VEGF-targeted agents including neutralizing antibodies to VEGF/VEGFR, soluble VEGFR/VEGFR hybrids and tyrosine kinase inhibitors with selectivity for VEGFR^22^. VEGF-targeted agents, administered either as single agents or in combination with chemotherapy, have been shown to benefit the patients with advanced-stage malignancies. The effects of VEGF-targeted therapy were mainly divided into two paradigms, the inhibition of new blood vessel growth and normalization of the tumor vasculature which may contribute the therapeutic effects^23^,^24^,^25^,^26^.

Vaccines for VEGF or its receptor have been reported to induce the CTL-mediating cellular immunity as well as antibody-mediating humoral immunity against tumor^14^,^15^,^27^. Because VEGF is produced from normal vascular or interstitial cells, and its receptors are widely expressed in endothelial cells, enhanced CTL-mediating cellular toxicity for long term may induce the side effect in terms of safety issue such as hypertension, myocardial and cerebrovascular infarction. Therefore, we designed epitope vaccine to activate humoral immunity, but not cellular immunity for VEGF. First, we examined the most appropriate epitope to either inhibit or neutralize the activity for VEGF-A. The candidate epitopes were three peptides: 7, 13 and 17 amino acid peptide, which contained the binding epitope of VEGF for bevacizumab and the binding region of VEGFR-1 or VEGFR-2^18^,^28^. From the present study, we finally selected 13 a.a. sequence to produce high anti-VEGF antibody. To assess the validity of the epitope (13 a.a. in VEGF-A), we also performed T-cell proliferation assay and ELISpot assay. In immunized female BALB/c mice, T-cell proliferation assay showed that the stimulation with VEGF 13 a.a. did not induce the proliferation of splenocytes from immunized mice, but rHBc and HBc epitope induced the proliferation of splenocytes (SI. 4). Similarly, in the ELISpot assay, the stimulation with VEGF 13 a.a. induced the production of neither IFN-γ nor IL-4, but stimulation with rHBc or HBc epitope did (SI. 5). In addition, the production of IFN-γ by rHBc or HBc epitope was by far more than IL-4, which also indicated that this immunization could lead to Th1-biased immune responses, along with the results of IgG subtype distribution. These data demonstrated that VEGF 13 a.a. did not induce T-cell activation, but HBc did, suggesting that HBc worked as an epitope carrier.

In this study, we designed VEGF DNA vaccine for alternative of the present anti-VEGF antibody therapy. Especially, we focused on induction of neutralizing antibody, but not cytotoxic T-cell activation, as HBc has ‘non-self’ helper T cell epitopes to elicit the potent B-cell and T-cell response in primates, and enhanced the immunogenicity of the epitope. Interestingly, anti-VEGF antibody induced by VEGF DNA vaccination showed *in vitro* neutralizing activity for ERK phosphorylation and tube formation in HUVECs and chemotactic activity in colon 26 cells. In vivo evidence also showed the inhibition of primary tumor growth through the inhibition of the number of CD31-positive vessels and the prolonged survival in tumor-bearing mice. From these results, combination with chemotherapy might be useful for VEGF DNA vaccination to enhance the therapeutic effects. Toward to the clinical trial, it might be considered for effective vaccination protocol, because our present study reflected the prevention model. It might be necessary to examine the therapeutic model to consider the clinical practice.

Overall, the present data first provided the potential activity to inhibit angiogenesis in cancer models, oxygen-induced retinopathy and laser-induced CNV models using DNA VEGF vaccine. DNA vaccine targeting mini-gene of VEGF might provide new therapeutic option in combination with chemotherapies or immunotherapies. We anticipate that the modification of this VEGF DNA vaccine will increase its potential clinical utility for the treatment of angiogenesis-related diseases such as cancer.

## Methods

### Construction of HBc-mVEGF fusion gene expression vector

We used the plasmid pcDNA3.1 (pcDNA3.1/V5-His-TOPO, Invitrogen) containing the cytomegalovirus promoter. The constructs were verified by nucleotide sequencing, by mRNA expression on RT-PCR and by protein expression on Western blots following transient transfection into COS-7 cells (SI. 1c).

### Animals and vaccination protocol

The experiments were approved by the Ethical Committee for Animal Experiments of the Osaka University Graduate School of Medicine. The mice had free access to water and food during the experimental periods. Five-week-old female BALB/c mice were purchased from CLEA Japan, Inc. and were acclimated for 1 week. Female BALB/c mice were vaccinated intramuscularly three times at 2-week intervals (6, 8 and 10 weeks old) with 60 μL of TE containing 120 μg of plasmid DNA or saline using an electric pulse generator with a pair of stainless steel needles that were 10 mm in length and 0.3 mm in diameter with a fixed distance between them of 3 mm (NEPA GENE). The voltage remained constant at 70 V during the pulse duration. Three pulses at the indicated voltage followed by three more pulses of the opposite polarity were administered to each injection site at a rate of one pulse/s, with each pulse being 50 ms in duration. Four weeks after the third immunization (14 weeks old), an additional immunization was administered to the mice.

### Measurement of anti-mVEGF antibody in serum

Eight or sixteen weeks after first immunization, serum was collected from the immunized mice of all groups. Serum levels of anti-VEGF antibodies in these mice were measured by ELISA. Briefly, ELISA plates were coated with 5 μg/mL VEGF 13 a.a. peptide conjugated BSA in carbonate buffer overnight at 4°C. Serial dilutions (1:100 to 1:312500) of serum samples from the immunized mice were added to the wells, and HRP-conjugated mouse IgG (whole IgG; GE Health care, UK, each subtype; Abcam, UK) was added. After four washes with PBST, 3,3′,5,5′-tetramethylbenzidine (TMB, Sigma-Aldrich, USA) was added. Production of the blue reaction product was stopped by adding 0.5 mol/L sulfuric acid, and the resulting end product (yellow) was read at 450 nm.

### T-cell proliferation assay and enzyme-Linked ImmunoSpot (ELISpot) assay

The T-cell proliferation assay was performed as previously reported. Cultured splenocytes from mice immunized with the HBc-mVEGF (13 a.a.) vaccine plasmid DNA either were not stimulated or stimulated with peptides containing the antigen sequence (mVEGF 13 a.a.; IMPIKPHQSQHIGE), recombinant HBc protein (rHBc), peptides containing the HBc CTL epitope, or phytohemagglutinin (PHA, 50 μg/mL, as positive control). Syngeneic T cells (mouse splenocytes, 5 × 10^5^ cells/well) were cultured with several stimulants at 37°C in 5% CO_2_ for 40 hours. Furthermore, 1 μCi of [^3^H] thymidine (Perkin Elmer) was added to each well for 8 hours. The cells were harvested, and the [^3^H] thymidine uptake (cpm) was determined using a MicroBeta 1450 Trilux scintillation counter (Wallac Oy). The stimulation index was expressed as the ratio of stimulated cells to non-stimulated cells.

The ELISpot assay was carried out using the Mouse IFN-γ Development Module and the Mouse IL-4 Development Module for their respective targets (R&D Systems, USA) according to the manufacturer's instructions.

### IgG purification

Mouse serum was purified using by Protein G column according to the manufacturer's instructions (MAbTrap Kit; GE Healthcare, UK). Briefly, serum samples were adjusted to the composition of the binding buffer (20 mM sodium phosphate, pH 7.0). The Protein G column was washed out the ethanol preservative with distilled water and equilibrated with binding buffer. The diluted serum was applied and the column was wash with binding buffer until no material appears in the effluent. After that, IgG fraction was eluted by elution buffer (0.1 M glycine-HCl, pH 2.7) and concentrated by centrifugal filter (Amicon Ultra; Millipore, USA).

### Tube formation assay

The tube formation assay using Matrigel (Becton-Dickinson, USA) was performed according to manufacturer's instructions. Briefly, matrigel was used to coat the wells of 24-well plates (0.25 mL per well) and was left to polymerize at 37°C for 1 hour. After polymerization, HUVECs (100,000 cells) suspended in 0.3 ml of EBM-2 (containing 20% EGM-2 kit, 0.4% FBS and 20% supplement) with control serum or immunized serum, were added to each well. After 6 hours, the wells were photographed at 50-fold magnification in five randomized fields (Olympus) and the number of their tubular networks was counted by using ImageJ.

### Mouse tumor model

Colon 26 parental colon carcinoma cell line was generously gifted by the laboratory of Dr. Nobuyuki Takakura, and cells were cultured in RPMI1640 medium containing 10% FBS. 1 × 10^6^ cells of colon 26 in 100 μL PBS were inoculated subcutaneously into BALB/c mice in the right side of back [n = 12 for HBc-mVEGF (13 a.a.) group, n = 3 for HBc group or n = 3 for saline group]. The tumor volumes were calculated in mm^3^ as 0.5 × (length) × (width)^2^. Survival was analyzed by the Kaplan-Meier method and compared between groups by use of the log-rank test.

### Immunohistochemistry

Tumors were removed at 12 days after s.c. inoculation of colon 26, fixed in 4% paraformaldehyde overnight, and equilibrated in phosphate buffer (PB) containing 15% sucrose for 2 days, and in PB/30% sucrose for 2 days. The samples were embedded for rapid freezing. Cross sections were laid on slides and stained; some sections were frozen at −80°C. For staining of microvessels, tissue sections air-dried at room temperature for 30 min, were blocked with 3% normal goat serum/PBST for 30 min and incubated overnight at 4°C with anti-mouse CD31 antibody (BD PharMingen, USA). Sections then were incubated with Alexa 488-conjugated anti-rat IgG (Molecular Probes, USA) and counterstained. The microvessel density was evaluated by counting CD31-positive vessels using fluorescence microscope (Olympus, Japan) at 400-fold magnification in randomly selected fields at the periphery of each section.

### Oxygen induced retinopathy (OIR)

OIR was induced in newborn mice as previously described by Smith et al. and modified by our group^29^,^30^. Briefly, newborn mice were exposed to hyperoxia (75 ± 0.5% O_2_) from P7 to P12 and returned to normoxia condition. At P14, we intravitreally injected 1 μl of PBS or purified mouse IgG of serum from mice immunized with HBc-mVEGF, HBc or saline into the right eyes of mice (*n* = 6 per group). At P17, mice were sacrificed with CO_2_ gas inhalation and the eyes were enucleated. The enucleated eyes were fixed with 4% paraformaldehyde and processed for further analyses. For qualitative analyses, fluorescein isothiocyanate dextran (500 kDa; Sigma-Aldrich) was intravenously injected 1 hour before the sacrifice. From the enucleated eyes, the retinal tissues were prepared, flat-mounted, and observed via the fluorescence microscope (BX50; Olympus). For quantitative analyses, the enucleated eyes were prepared for paraffin sections. Quantitative analysis was performed by direct counting of vascular lumens on the vitreal side of the inner limiting membrane in at least 10 sections from each eye (*n* = 6 per group).

### Laser-induced CNV

CNV lesions were induced in the right eyes of mice by laser photocoagulation as previously described^31^. Briefly, 6-week-old male C57BL/6 mice were anesthesized and the pupils were dilated with 1% tropicamide (Alcon Laboratories, USA). Laser photocoagulation was performed to each 3, 6, 9, and 12 o'clock position of 2 disc diamters from the optic disc by using the indirect head set delivery system (Oculight; Iridex, USA) and a handheld +78 diopter lens. On the 10th day after the laser photocoagulation, we intravitreally injected 1 μl of PBS or purified mouse IgG of serum from mice immunized with HBc-mVEGF, HBc or saline into the right eyes of mice (*n* = 6 per group). After additional 4 days, the mice were sacrificed with CO_2_ gas inhalation and the eyes were enucleated to investigate the antiangiogenic effect of tested molecules on choroidal neovascularization. For quantitative analyses, the enucleated eyes were prepared for immunofluorescence staining. After 1 hour fixation with 4% paraformaldehyde, the retinal pigment epitheium (RPE)-choroid-sclera complex was prepared. The RPE-choroid-sclera complex was washed with cold buffer (1% Triton X-100, 0.1 mM CaCl_2_·2H_2_O, 0.1 mM MgCl_2_, and 0.1 mM MnCl_2_·4H_2_O) in pH 6.8 phosphate-buffered saline (PBS), treated with 1:10 Isolectin GS-IB4 conjugated with Alexa Fluor 568 (Invitrogen, USA) overnight, and washed 3 times with PBS. Then, flat-mounted RPE-choroid-sclera complex was evaluated with the fluorescence microscopy (BX50; Olympus).

### Statistical analysis

Results were subjected to statistical analysis. Survival curves were analyzed using Kaplan-Meier method with log-rank test. Student's *t* test was two-tail and unpaired.

Detailed information is described in the supplementary information.

## Author Contributions

M.K., H.N., N.T. and R.M. designed research; M.K., H.K., H.T., F.N., M.S., H.K. and P.Z. performed the experiments and prepared Figure 1–3. D.H.J. and J.H.K. prepared Figure 4. M.K. and H.N. wrote the paper. All authors discussed and agreed on the results.

## Supplementary Material

Supplementary Information Supplement Figures
