# Supplementary material for: Development of novel DNA vaccine for VEGF in murine cancer model
Source: Sci Rep. 2013 Nov 29;3:3380. doi: 10.1038/srep03380 (PMC3843165; doi:10.1038/srep03380)
Supplement: Supplementary Information — Supplement Figures [file srep03380-s1.pdf]

Supplemental Information

Development of novel DNA vaccine for VEGF in murine cancer model

Mariko Kyutoku[1,2], Hironori Nakagami[2], Hiroshi Koriyama[1,2], Hideki Tomioka[1,2], Futoshi Nakagami[1,2], Munehisa Shimamura[2], Hitomi Kurinami[2], Pang Zhengda[2,3], Dong Hyun Jo[4,5], Jeong Hun Kim[4,5,6], Nobuyuki Takakura[7], and Ryuichi Morishita[1]

[1]Department of Clinical Gene Therapy, Osaka University Graduate School of Medicine
[2]Division of Vascular Medicine and Epigenetics, Department of Child Development, United Graduate School of Child Development, Osaka University, Kanazawa University, Hamamatsu University School of Medicine, Chiba University and Fukui University
[3]Department of Geriatric Medicine, Osaka University Graduate School of Medicine
[4]Fight against Angiogenesis-Related Blindness (FARB) Laboratory, Clinical Research Institute, Seoul National University Hospital, Seoul, Republic of Korea
[5]Department of Biomedical Sciences, College of Medicine, Seoul National University, Seoul, Republic of Korea
[6]Department of Ophthalmology, College of Medicine, Seoul National University, Seoul, Republic of Korea
[7]Department of Signal Transduction, Research Institute for Microbial Diseases, Osaka University, Suita, Osaka, Japan
[8]JST(Japan Science and Technology Agency), CREST, Tokyo, Japan

Corresponding authors

Ryuichi Morishita, M.D., Ph.D.
Department of Clinical Gene Therapy,
Osaka University Graduate School of Medicine
2-2 Yamada-oka Suita 565-0871, Japan
Tel: 81-6-6879-3406, Fax: 81-6-6879-3409
E-mail: morishit@cgt.med.osaka-u.ac.jp

and

Hironori Nakagami, M.D., Ph.D.
Division of Vascular Medicine and Epigenetics,
Osaka University Graduate School of Child Development
2-1 Yamada-oka Suita 565-0871, Japan
Tel: 81-6-6879-4142, Fax: 81-6-6879-4142
E-mail: nakagami@cgt.med.osaka-u.ac.jp

Supplemental Information 1

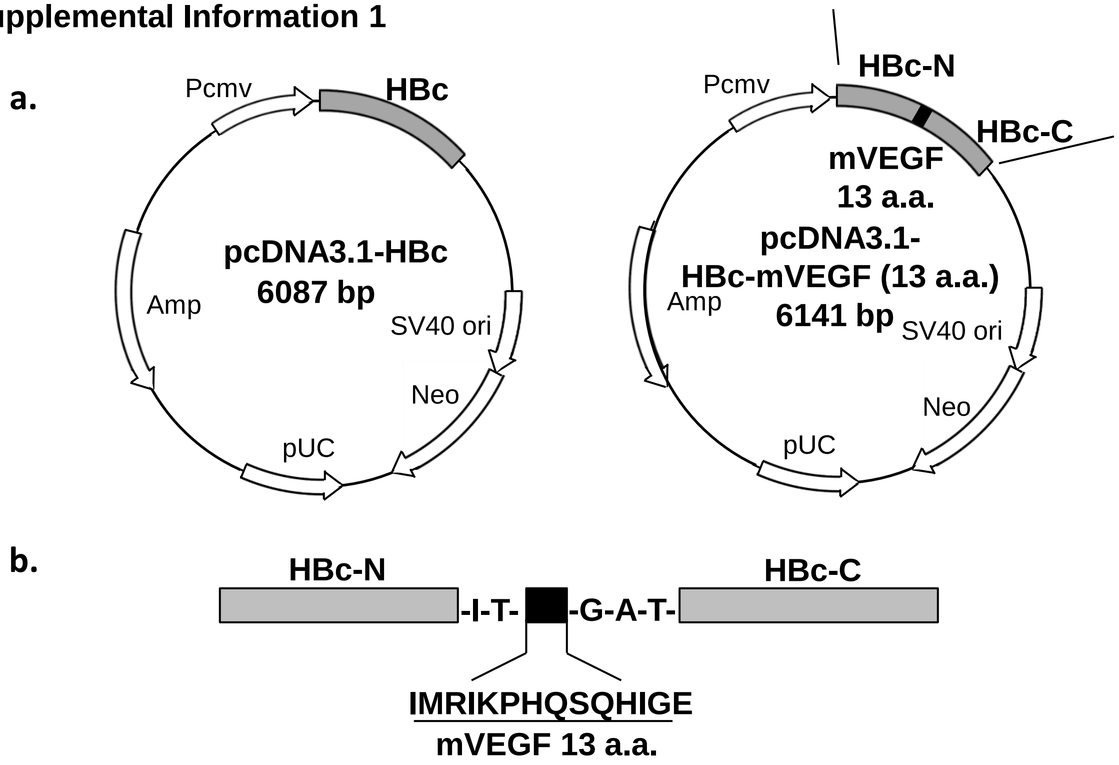

Supplemental information 1. Plasmid DNA construction of the vaccine

a) Plasmid maps of pcDNA3.1-HBc (control vector) and pcDNA3.1-HBc-mVEGF (13 a.a.) (vaccination vector). HBc indicates the full sequence of HBc, HBc-N indicates the N-terminus of HBc (1-80 a.a.), and HBc-C indicates the C-terminus of HBc (81-183 a.a.). mVEGF 13 a.a. indicates the antigen for the mouse VEGF protein, as mentioned below.

b) Detail information regarding the VEGF vaccine plasmid design is shown. Thirteen amino acids (IMRIKPHQSQHIGE), which served as an antigen for VEGF and the linkers (the N-terminal I-T dipeptide linkers and C-terminal GAT tripeptide), were designed fuse in-frame to VEGF to allow for flexibility in the conformation of the VEGF epitope when the surface is exposed on the HBc particle. The VEGF 13 a.a. and linkers are represented by single-letter codes.

Supplemental Information 2

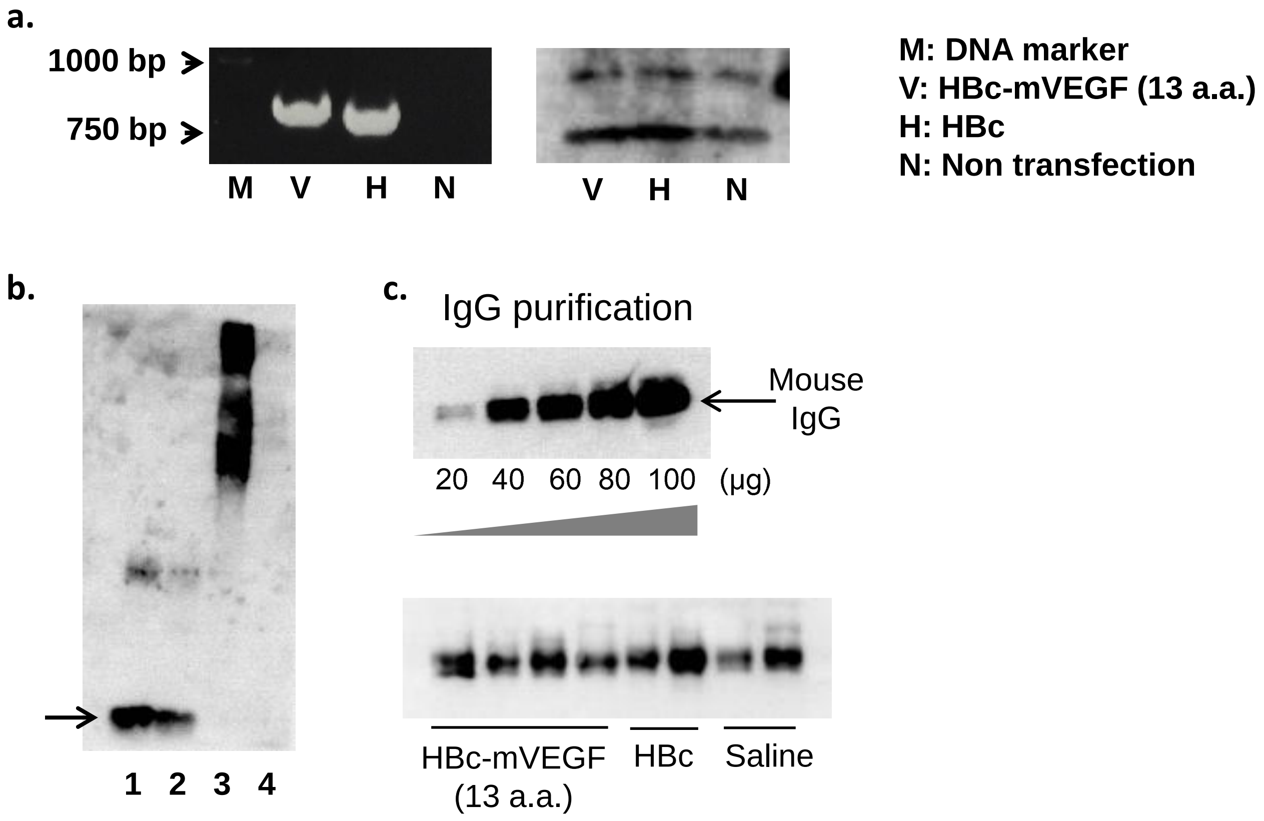

Supplemental information 2. Confirm of DNA construction and purified IgG

a) Expression of plasmid DNA constructs in transfected COS-7 cells. mRNA and protein expression levels of HBc-mVEGF (13 a.a.) were assessed by RT-PCR and western blot analysis, respectively, after transient transfection of each vector into COS-7 cells. Each letter indicates the following plasmid; V, HBc-mVEGF (13 a.a.); H, HBc; N, No transfection.

b) Specific binding of immunized serum to VEGF. Immunized serum was used as primary antibody in western blot, and it bound to BSA-conjugated mVEGF (13 a.a.), recombinant mouse VEGF-A (rmVEGF), and recombinant human VEGF-A (rhVEGF). Loading samples were as follows: lane 1, rmVEGF; lane 2, rhVEGF; lane 3, BSA-conjugated mVEGF (13 a.a.); lane 4, BSA-conjugated human angiopoietin-2 peptide (negative control).

c) IgG purification from mouse serum. The IgG fraction isolated form purified serum was confirmed by western blotting, referenced to commercial mouse IgG. The blotted membrane was directly incubated with HRP-conjugated anti-mouse IgG and visualized.

Supplemental Information 3

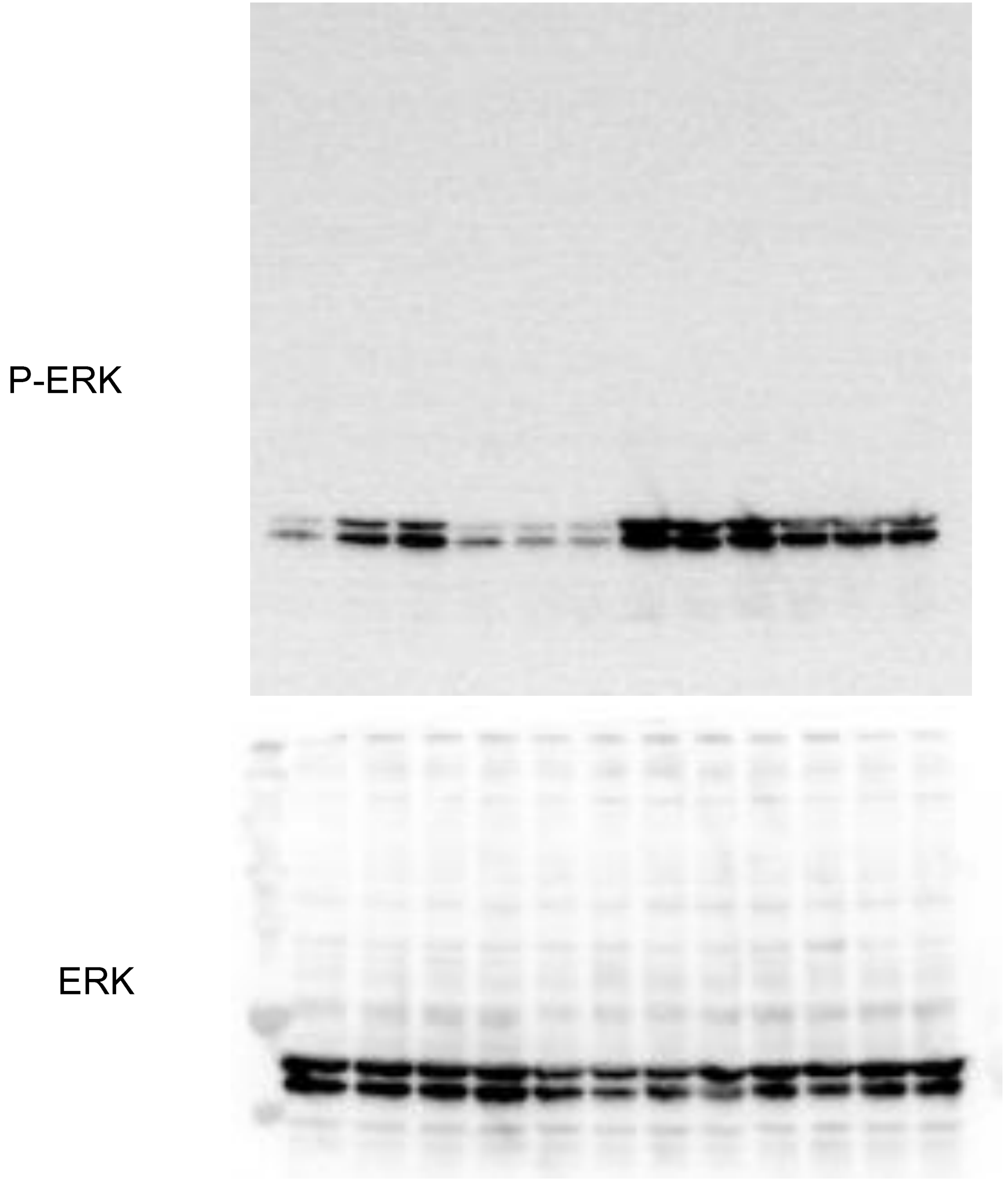

**mVEGF (5 ng/mL)**	-	+	+	-	-	-	+	+	+	+	+	+
**Immunized serum**	-	-	-	-	-	-	-	-	-	+	+	+
**Control serum**	-	-	-	-	-	-	+	+	+	-	-	-
**Elution & Neutralizing buffer**	-	-	-	+	+	+	-	-	-	-	-	-

Supplemental information 3. Neutralizing activity of immunized serum in HUVECs
a) Western blot analysis of cell lysates from HUVECs stimulated for 10 min with mVEGF at 5 ng/mL in the presence of immunized serum or control serum for p-ERK and total ERK.

Supplemental Information 4

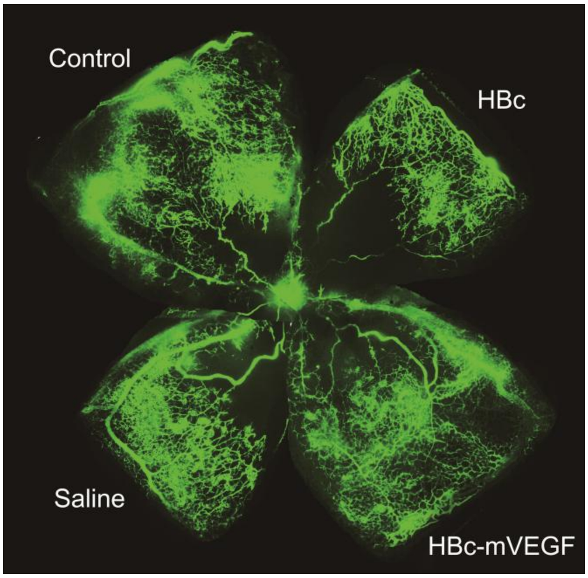

Supplemental information 4. Qualitative analysis of the mouse OIR model. Purified IgG from immunized serum was intravitreally injected into the right eye of mice at P14, and the enucleated eyes were prepared for further analyses at P17. For qualitative analyses, FITC-dextran (500 kDa) was intravenously perfused, and the retinal tissues were prepared and flat-mounted. Images from each group were intentionally modified and combined to demonstrate the inhibitory effect of the purified IgG on the formation of vascular tufts.

Supplemental Information 5

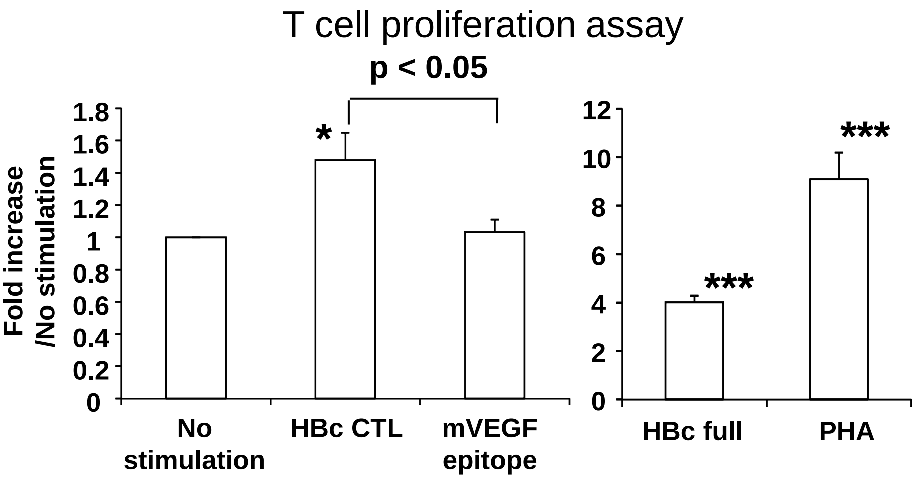

Supplemental information 5. T-cell proliferation assay to DNA vaccination

T-cell proliferation assays were performed by measuring [^{3}H] thymidine uptake. Cultured splenocytes from mice immunized with the HBc-mVEGF (13 a.a.) vaccine plasmid DNA either were not stimulated or stimulated with peptides containing the antigen sequence (mVEGF 13 a.a.; IMPIKPHQSQHIGE), recombinant HBc protein (rHBc), peptides containing the HBc CTL epitope, or PHA. Each HBc protein was used as a positive control for specific T-cell activation, and PHA was used as a positive control for non-specific T-cell activation.

Supplemental Information 6

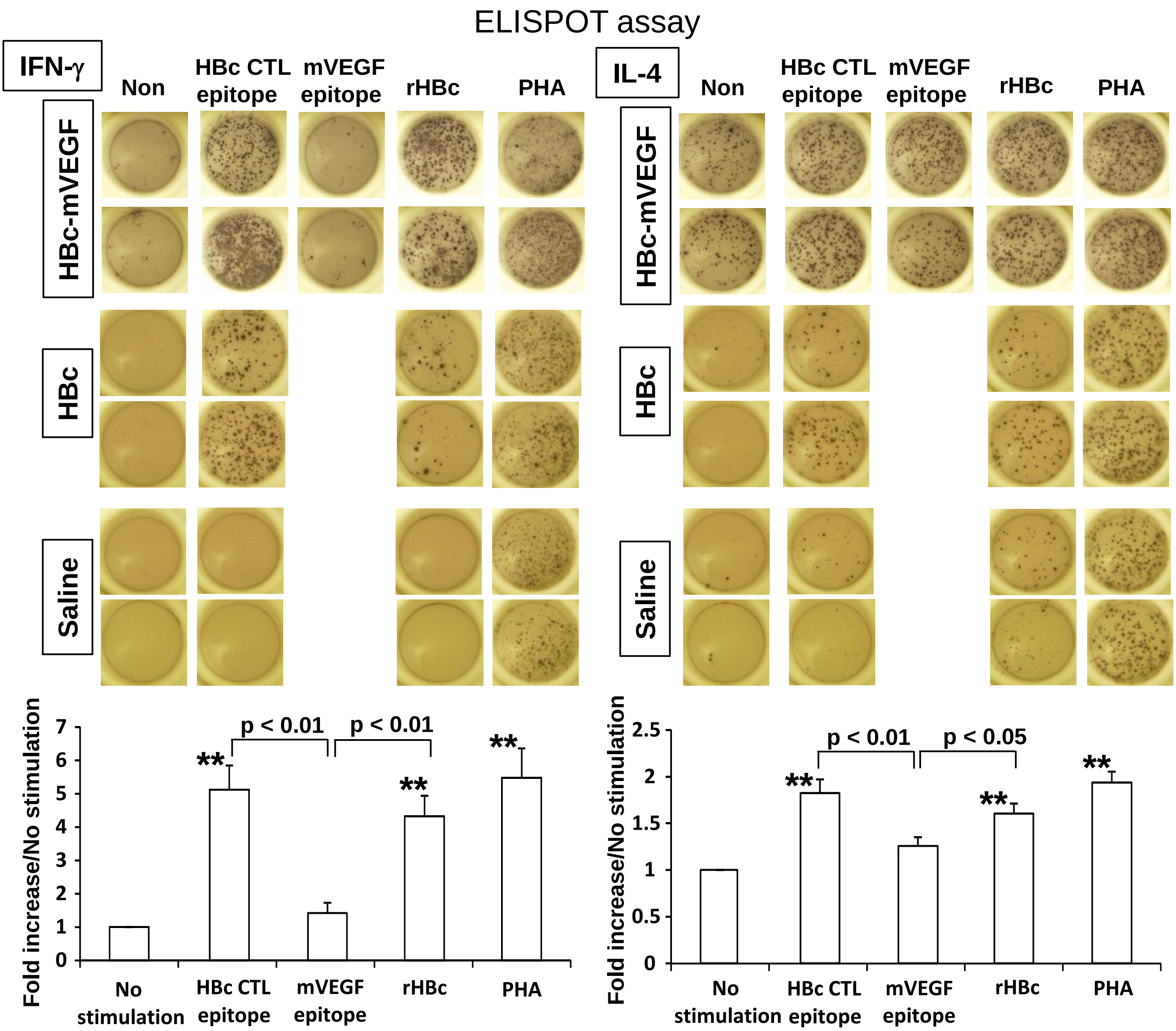

Supplemental information 6. ELISpot assay to DNA vaccination

ELISpot assay. Splenocytes were obtained from the HBc-mVEGF (13 a.a.), HBc, or saline groups, and they either were stimulated or not stimulated with peptides containing the antigen sequence, rHBc, peptides containing the HBc CTL epitope, or PHA. The blue dots are positive for IFN-γ (left panel) and IL-4 (right panel). Positive cells were quantified by counting the number of spots in each well, and more than three wells per group were counted. Each HBc protein was used as a positive control for specific T-cell activation, whereas PHA was used as a positive control for non-specific T-cell activation.
